# Co-immobilization of an Enzyme and a Metal into the Compartments of Mesoporous Silica for Cooperative Tandem Catalysis: An Artificial Metalloenzyme**

**DOI:** 10.1002/anie.201306487

**Published:** 2013-11-12

**Authors:** Karin Engström, Eric V Johnston, Oscar Verho, Karl P J Gustafson, Mozaffar Shakeri, Cheuk-Wai Tai, Jan-E Bäckvall

**Affiliations:** Department of Organic Chemistry, Arrhenius Laboratory, Stockholm University10691 Stockholm (Sweden); Department of Materials and Environmental Chemistry and Berzeli Center EXSELENT on Porous Material, Arrhenius Laboratory, Stockholm University10691 Stockholm (Sweden)

**Keywords:** artificial deracemase, biocatalysis, dynamic kinetic resolution, hybrid catalysts, metal catalysis

As a result of natural evolution for millions of years, enzymes constitute the state of the art in catalysis, with their ability to efficiently catalyze a wide range of important chemical reactions under mild conditions. Among the enzymes, metalloenzymes stand out as the most impressive examples, and they are involved in several important processes in nature, such as water oxidation,[1] photosynthesis,[2] and nitrogen fixation.[3] In a metalloenzyme, a metal-containing cofactor is integrated into the protein structure to enable new chemical reactions that would not be possible to achieve with the chemistry of the protein alone. In modern society, the utilization of enzymes for large-scale production of chemicals and other industrial applications is well-established.[4] Along with the high efficiency of enzymes, they are also ideal sustainable catalysts, as they are obtained from renewable sources, and are biodegradable and non-toxic.

Unfortunately, one major drawback of naturally derived enzymes is that they often exhibit a narrow substrate scope, which limits their use in organic synthesis. Ever since the visionary report by Wilson and Whitesides in 1978,[5] a long-sought dream has been to construct metalloenzymes with non-natural cofactors that open up for new efficient catalytic reactions. Several approaches to generate artificial metalloenzymes have so far been explored,[6] but in general most of them involve the anchoring of an organometallic species to a protein, by either covalent, dative, or supramolecular interactions. In many of these cases the catalytic activity of the metalloenzyme originates from the introduced metal species, and the surrounding protein only serves as a ligand determining the stereoselectivty.

Recently, highly active heterogeneous catalysts, and in particular those based on nanometallic species, have attracted considerable attention for selective chemical transformations.[7] It has been found that by decreasing the cluster size below 2 nm a unique selectivity and activity may be obtained.[7a] Herein, we propose an approach for the construction of an artificial metalloenzyme with two different modes of reactivity: one exhibited by a nanometallic component and one by an enzyme. By the stepwise immobilization of an enzyme and a nanometal species into the same cavity of a mesoporous heterogeneous support, an environment is created where these species can reside and work in close proximity to one another in a cooperative fashion. This environment allows a simple design of cascade reactions in which the enzyme and the metal catalyst can work in cooperative tandem catalysis. Moreover, this approach would provide access to the advantages of heterogeneous catalysis with simple separation and recycling of the catalyst.

The approach of developing catalytic entities that make use of the potential of both biocatalysis and transition metal catalysis has so far only been sparingly explored. Foulkes et al. engineered *Escherichia coli* bacteria to simultaneously express monoamine oxidase intracellularly and bind Pd nanoparticles on the outer membrane, which were then used for amine deracemization.[8] Another promising strategy of generating multifunctional metalloenzyme mimics was recently reported by Kim and co-workers, where Pt nanoparticles were introduced into an aminopeptidase.[9] This artificial metalloenzyme was successfully employed in a two-step cascade reaction, involving enzyme-catalyzed amide bond cleavage and Pt-catalyzed hydrogenation. Very recently, the same approach was used by Filice et al. to introduce Pd nanoparticles into the interior of a lipase to obtain a biohybrid catalyst.[10]

In our group, recent research has been devoted to exploring siliceous mesocellular foams (MCFs)[11] as a support for various catalytic species.[12] These mesoporous silica materials are simple to prepare and they possess many desirable properties, such as large pore volumes, large surface areas, and they enable for high loadings of catalytic species that are shielded from mechanical grinding and leaching. The material also has a high surface concentration of silanol groups that allow grafting of various functional groups.

**Scheme 1:**
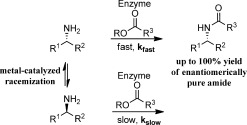
The principle of a dynamic kinetic resolution (DKR) of a primary amine.

To demonstrate the power of this co-immobilization method, the dynamic kinetic resolution (DKR) of a primary amine[13] was chosen as model reaction (Scheme 1). In this reaction a lipase enzyme acts as a resolving agent that selectively transforms only one enantiomer of the starting amine into the corresponding amide. To be able to convert the unreacted enantiomer into product and ensure a maximum theoretical yield of 100 % together with a high enantioselectivity, the resolution process is coupled to an in situ racemization of the amine performed by another catalytic species. This racemization reaction allows interconversion of the amine enantiomers and continuously feeds the enzyme-catalyzed resolution with more of the reactive enantiomer.

Recently, we reported on the immobilization of *Candida antarctica* lipase A (CALA) in MCF and its application in the kinetic resolution (KR) and DKR of β-amino esters employing either Shvo’s catalyst[14] or Pd/AlO(OH)[15] as a racemization agent.[12c,d] In a subsequent study, Pd nanoparticles were immobilized into the MCF, to give a highly efficient racemization catalyst for primary amines.[12b] With these two MCF-based procedures in hand, we envisioned that it should be possible to combine them and co-immobilize the metal and the lipase in MCF so that each cavity of the support will contain both the lipase and the nanopalladium. This co-immobilization would give a single and recyclable hybrid catalyst with the ability to perform both KR and racemization within the same cavity, thus fulfilling the requirements for an ideal DKR.

In this hybrid catalyst, each pore could be visualized as an artificial metalloenzyme, containing both amino acid constituents in the form of the immobilized lipase and a metal cofactor in the form of Pd nanoparticles. Moreover, the overall catalytic function of this artificial metalloenzyme, that is, deracemization, would constitute a reaction type that is unprecedented in nature.

**Scheme 2:**
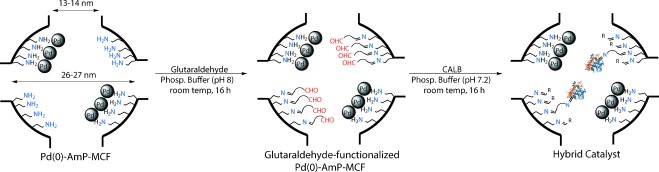
Synthetic strategy for the construction of the hybrid catalyst. In the first step the free amine groups of the Pd^0^-AmP-MCF are post-functionalized with a dialdehyde linker by a Schiff-base forming reaction. In the final step, the other end of the supported dialdehyde is used as a handle for the anchoring of the enzyme to the support by yet another Schiff-base forming reaction.

As the lipase constituent we chose *Candida antarctica* lipase B (CALB), which has been previously used in DKR of primary amines.[13] As depicted in Scheme 2, the synthesis of a Pd-CALB hybrid catalyst was carried out starting from our previously developed Pd^0^-AmP-catalyst.[12a,b] In the latter catalyst, Pd nanoparticles are immobilized in aminopropyl-functionalized MCF, and we hypothesized that it should be possible to chemically modify the aminopropyl groups so that they could accommodate an enzyme. This approach was thought to be more practical compared to immobilizing the enzyme prior to the introduction of the Pd nanoparticles, as it was expected that the enzyme would be damaged by the harsh and strongly basic conditions necessary for the generation of the Pd nanoparticles.

Calculations based on elemental analysis data for the Pd^0^-AmP-MCF nanocatalyst, showed that the Pd:N ratio was about 1:1.4, suggesting that there are plenty of free aminopropyl groups that are uncoordinated to Pd and that could be further functionalized. Post-functionalization of the remaining aminopropyl groups was carried out by treating the Pd^0^-AmP-MCF with aqueous glutaraldehyde in phosphate buffer (pH 8). In this reaction, one end of the dialdehyde is linked to a free amine of the support by a Schiff-base forming reaction. CALB was then covalently linked to the support by another Schiff-base forming reaction (in phosphate buffer at pH 7.2), between the other end of the dialdehyde linker and a lysine or an arginine residue situated on the enzyme surface. Different loadings of glutaraldehyde (0.1, 0.5, and 2.0 equiv with respect to the aminopropyl groups on the support) and CALB (5.5 wt % and 17 wt % of enzyme with respect to the Pd^0^-AmP-MCF) were screened and the resulting hybrid catalysts were evaluated in both amine racemization and KR of 1-phenylethylamine. The racemization activity of the Pd nanoparticles was found to be highly dependent on the amount of glutaraldehyde used in the post-functionalization (Supporting Information, Figures S2–S7 and Tables S1–S6). With 0.1 equiv of glutaraldehyde, 75 % of the racemization activity was preserved, whereas with 0.5 and 2.0 equiv only 49 % and 16 %, respectively, of the racemization activity was maintained (SI, Figure S7 and Table S6). Control experiments with Pd^0^-AmP-MCF (no enzyme) with varying amounts of immobilized glutaraldehyde showed a similar trend, where a decrease in reaction rate was seen upon increasing amounts of glutaraldehyde (SI, Table S7).

Surprisingly, the KR activity of the hybrids seemed to be negligibly affected by the amount of glutaraldehyde employed in the first step, and all hybrids prepared from 17 wt % CALB gave essentially the same conversion and enantiomeric excess (*ee*) after 1 h at 70 °C (SI, Table S8, entries 1, 3, and 4).[16] These observations suggest that 0.1 equiv of glutaraldehyde is sufficient for immobilizing the required amount of enzyme for an efficient KR. Lowering the amount of CALB to 5.5 wt % on the other hand, resulted in a significantly slower KR and only 18 % of the acylated product was obtained after 1 h (Table S8, entry 2). Interestingly, the racemization rate did not improve when the enzyme loading was lowered (SI, Figure S7, Table S6), which clearly demonstrates that this co-immobilization strategy keeps the two species sufficiently separated to avoid deactivating interactions between them. Based on the results obtained from the racemization and KR, the hybrid prepared from 0.1 equiv glutaraldehyde, and 17 wt % CALB, hereafter denoted as hybrid-GA_0.1_-E_high_, was used for characterization and DKR experiments.

From elemental analyses (ICP-OES), the loading of enzyme and Pd on the hybrid-GA_0.1_-E_high_ was determined to be 15.6 wt % and 4.80 wt %, respectively. These data confirm that the glutaraldehyde-based method for immobilization of CALB on the support was highly efficient, as 92 % of the loaded enzyme was successfully attached. Another piece of desirable information is the distribution of CALB on the support. However, direct observation of the enzyme by transmission electron microscopy (TEM) is not possible. Therefore, a method was developed that involved tagging of CALB with gold nanoparticles to allow for the indirect determination of the enzyme distribution (SI, Figure S1).

**Figure 1:**
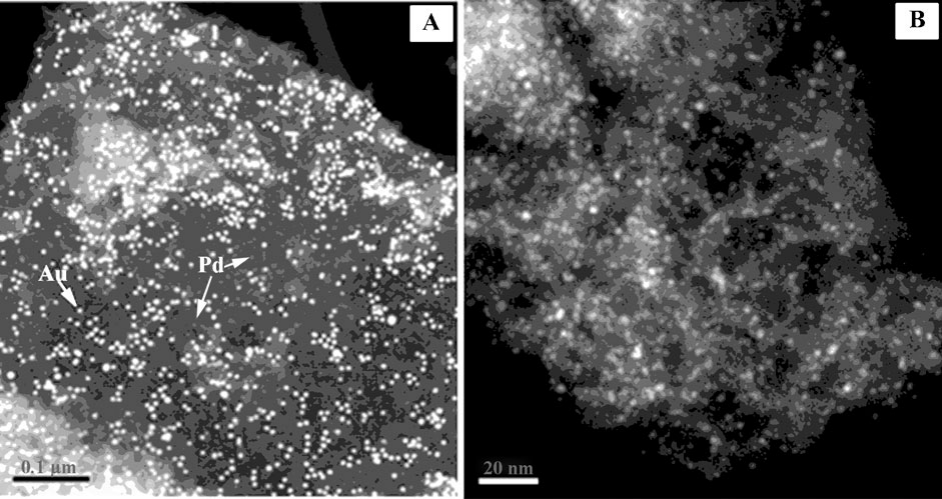
A) Image of the hybrid catalyst containing 5 nm Au-tagged CALB by HAADF-STEM, showing two sets of well-dispersed metal nanoparticles. The larger and brighter set of nanoparticles belongs to the Au tags that confirm a uniform distribution of the enzyme on the support. The smaller set with darker contrasts belongs to the original Pd nanoparticles, which are responsible for the racemization activity. Scale bar: 0.1 μm. B) HAADF-STEM image of the corresponding hybrid catalyst without Au nanoparticles tags for clearer a view of the Pd nanoparticle distribution. The typical size range of the Pd nanoparticles was found to be 1–2 nm. Scale bar: 20 nm.

To provide a sufficient Au nanoparticle tagging, a solution of CALB was first treated with tris(2-carboxyethyl)phosphine (TCEP), which reductively cleaves a disulfide bridge that is present on the enzyme surface into free thiols. Thiols are well-known to efficiently coordinate to Au nanoparticles, and this property was exploited when anchoring these nanoparticles to CALB. TCEP-treated CALB was stirred overnight with a colloidal solution containing either 2 or 5 nm Au nanoparticles, followed by concentration of the resulting reaction solution in vacuo. The solution of the 2 nm Au reaction was passed through a membrane centrifugation tube with a cutoff of at 10 K prior to concentration. Elemental analysis (ICP-OES) of the collected enzyme residue was for practical reasons only possible for the 2 nm reaction, and it showed that 14 % of the CALB had been successfully tagged with an Au nanoparticle. This was considered to be sufficient for subsequent TEM experiments. The tagged enzyme was then immobilized into the glutaraldehyde-functionalized Pd^0^-AmP-MCF as described above and analyzed by high-angle annular dark-field scanning transmission electron microscopy (HAADF-STEM, Figure 1 A; SI, Figures S8,9). To our delight, a well-dispersed pattern of Au nanoparticles of either 2 nm or 5 nm could be observed along with the Pd nanoparticles in both cases. For better visualization of the Pd nanoparticles with weaker contrast, additional TEM and STEM analyses of the corresponding hybrid catalyst in the absence of the Au nanoparticle tags were also conducted as a reference, which showed a well-dispersed Pd nanoparticle distribution in the size-range of 1–2 nm (Figure 1 B; SI, Figure S10). Furthermore, this demonstrates that our method of introducing glutaraldehyde and enzyme on the Pd^0^-AmP-MCF does not lead to any significant clustering or agglomeration of the Pd nanoparticles present from the start.

The utilization of hybrid-GA_0.1_-E_high_ as a catalyst for the DKR of 1-phenylethylamine was commenced by performing the reaction at 80 °C, as both the kinetic resolution and the racemization previously showed high efficiency at this temperature. However, it was found that these conditions were not ideal for the DKR, and only a moderate yield of the desired product **2** was obtained as a result of partial enzyme deactivation (Table 1, entry 1). The explanation for this seemingly contradictory result originates from the difference in reaction time between KR and DKR. In KR the reaction time is short with negligible deactivation, whereas in DKR the reaction time is long, leading to significant enzyme deactivation.

**Table 1:** Summary of the results from the DKR of 1-phenylethylamine catalyzed by the hybrid-GA_0.1_-E_high_ and the separate component system.[a] 
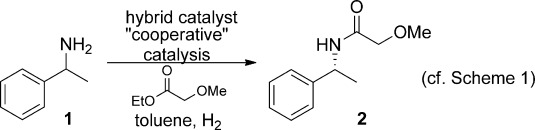

Entry	Catalyst	*t* [h]	*T* [°C]	Yield [%][b]	*ee* [%][b]
1	Hybrid-GA_0.1_-E_high_	24	80	45	99
2	Hybrid-GA_0.1_-E_high_	16	70	95	99
3	Hybrid-GA_0.1_-E_high_[c]	16	70	99	99
4	Pd^0^-MCF/CALB-MCF	16	70	66	99
5	Pd^0^-MCF/CALB-MCF[c]	20	70	89	99

[a] Reaction conditions: All of the reactions were carried out in toluene (2 mL) under 1 atm of hydrogen gas, 1-phenylethylamine (0.60 mmol), ethyl methoxy acetate (1.20 mmol), dry Na_2_CO_3_ (50 mg), and pentadecane as internal standard.

[b] Determined by GC analysis.

[c] Performed with 4 Å molecular sieves.

The problem of enzyme deactivation was circumvented by lowering the temperature to 70 °C, which furnished **2** in 95 % yield and an excellent enantioselectivity (99 % *ee*) after 16 h (Table 1, entry 2). The DKR employing the hybrid could be further improved by the addition of molecular sieves (4 Å), which afforded the desired product in quantitative yield and 99 % *ee* after 16 h (Table 1, entry 3).

**Figure 2:**
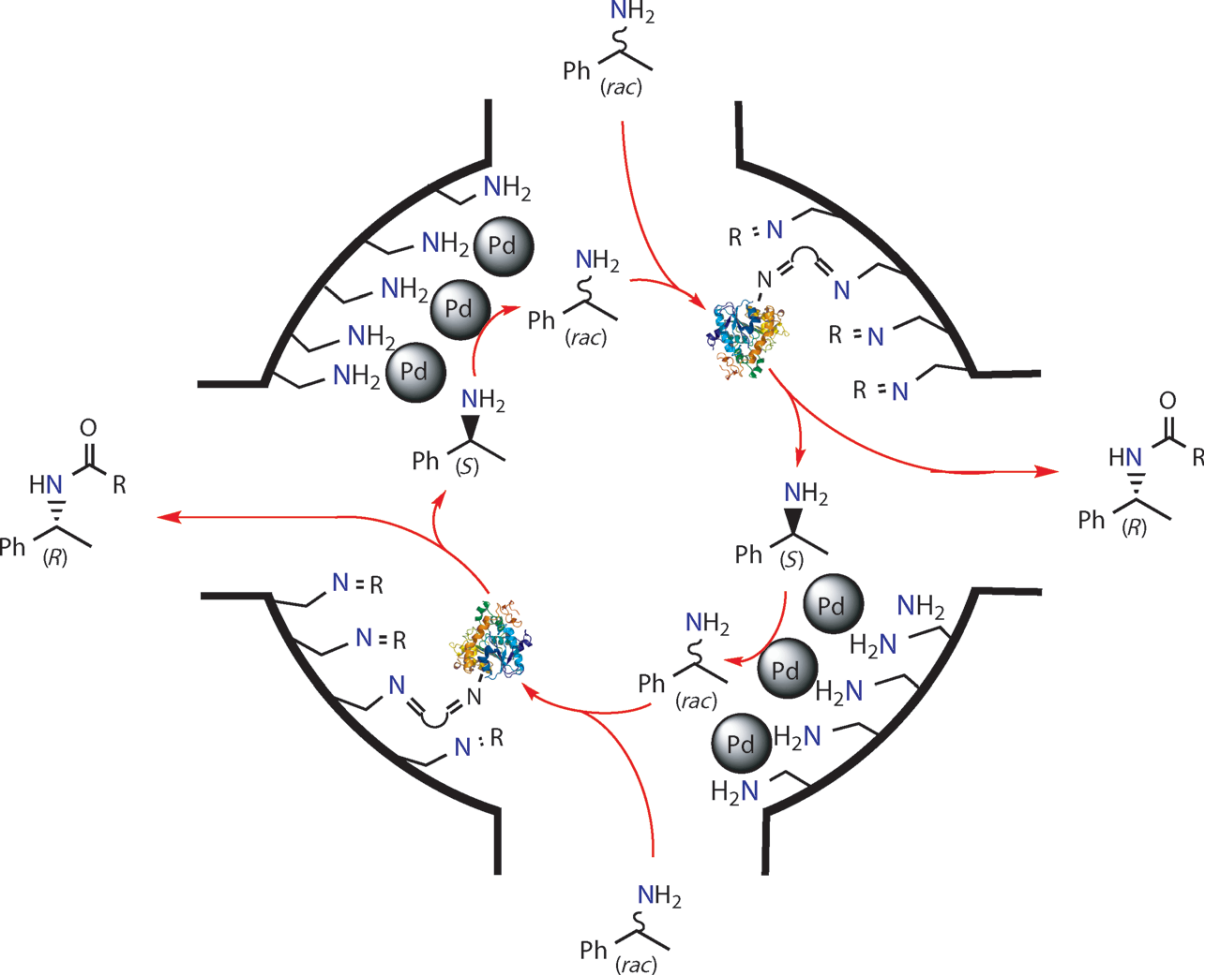
Dynamic kinetic resolution of an amine with a bifunctional biomimetic catalyst in which Pd nanoparticles and a lipase (CALB) are co-immobilized in the same pore of MCF.

To validate the utility of the concept of co-immobilizing the Pd nanoparticles and the enzyme into the same cavities of the support, a DKR was performed with separate Pd^0^-AmP-MCF and CALB-MCF (Table 1, entries 4,5). To make these results fully comparable, the respective loadings of Pd and enzyme used in the separate component reaction were the same as those employed in the hybrid-GA_0.1_-E_high_. Moreover, the Pd^0^-AmP-MCF used in this comparison was of identical standard to that used for the hybrid catalyst preparation, and the immobilization of CALB was done in exactly the same manner as that used for the enzyme immobilization in the hybrid catalyst. Interestingly, the separate component reaction displayed significantly lower efficiency than that of the hybrid catalyst, resulting in a yield of only 66 % after 16 h (Table 1, entry 4). By the addition of molecular sieves (Table 1, entry 5) the yield of the separate component reaction could be improved to 89 % (reaction time 20 h), but it was still significantly lower than the corresponding reaction of the hybrid catalyst (Table 1, entry 3). These results demonstrate that by co-immobilizing the Pd nanoparticles and CALB into the same cavities of the support, a substantially more efficient and robust DKR can be achieved. The higher efficiency of the hybrid reaction can be ascribed to the fact that each cavity contains the two catalysts and that this co-immobilization results in a more efficient cooperation between the two catalysts. The principle for this cooperative tandem catalysis within the MCF cavity is given in Figure 2. Racemic amine (*rac*) enters the cavity and is exposed to the enzyme, leading to a selective acylation of the (*R*)-enantiomer of the amine. The (*R*)-amide exits from the cavity, whereas the (*S*)-enantiomer of the amine will be racemized by the nanopalladium inside the cavity. The racemic amine (*rac*) formed will again undergo an enzymatic resolution, and the process is repeated.

An important aspect of heterogeneous catalysis is the recyclability, and consequently the evaluation of hybrid-GA_0.1_-E_high_ proceeded by employing it in consecutive cycles of the DKR of 1-phenylethylamine, both with and without molecular sieves (SI, Table S10, entries 1–5). Gratifyingly, it is possible to recycle the hybrid catalyst in both cases, although a decrease in efficiency could be observed over each cycle. However, by extending the reaction time of the second cycle, it was possible to obtain high yields and excellent *ee* values of the desired amide **2** (Table S10, entry 2), while for the third cycle this showed not to be possible, at least not within practical reaction times (Table S10, entry 3). We were able to ascribe this deactivation to the enzyme component, by examination of the *ee* of the starting amine, which revealed that the racemization catalyzed by the Pd nanoparticles proceeded efficiently throughout the reactions.

To establish the cause of the CALB deactivation in the hybrid, several control experiments were conducted. Leaching tests were conducted using ICP-OES, from which it was found that less than 5 % of the CALB had leached. This small leaching cannot account for the enzyme deactivation. ICP-OES analysis also showed that the Pd leaching was low (0.1 ppm). Therefore, it was proposed that denaturation of CALB is the reason for the observed deactivation. Loss of enzyme activity caused by leached Pd was ruled out based on recycling studies performed on the CALB-MCF (SI, Table S11), which showed similar deactivation on recycling. This suggests that the denaturation of CALB is triggered by the polar surface of the support, which has been observed previously for other enzymes.[17] In future work, other less-polar materials, such as mesoporous carbon or glass, will be explored in attempts to minimize the enzyme denaturation.

In summary, we have demonstrated herein that it is possible to develop a highly efficient multifunctional hybrid catalyst based on heterogeneous materials containing several fundamentally different catalytic species. In this hybrid catalyst, a metal and a lipase cooperate in an unprecedented fashion, which is best described as an artificial “deracemase” metalloenzyme. The hybrid catalyst was well characterized, confirming that nanopalladium and enzyme were immobilized into the same cavities of the mesocellular foam. Furthermore, it was demonstrated that the proximity of the two catalytic species conferred an enhanced efficiency in the dynamic kinetic resolution of an amine. The concept of co-immobilizing catalysts with different functions into mesoporous materials holds great promise for creating other hybrid catalysts with unprecedented reactivity. This will be important for the future development of catalytic entities that can operate by cooperative tandem catalysis for the chemical synthesis of the future.
