# Supplementary material for: Co-immobilization of an Enzyme and a Metal into the Compartments of Mesoporous Silica for Cooperative Tandem Catalysis: An Artificial Metalloenzyme
Source: Angew Chem Int Ed Engl. 2013 Nov 12;52(52):14006–10. doi: 10.1002/anie.201306487 (PMC4499256; doi:10.1002/anie.201306487)

Angewandte Chemie
Eine Zeitschrift der Gesellschaft Deutscher Chemiker

Supporting Information
© Wiley-VCH 2013
69451 Weinheim, Germany

Co-immobilization of an Enzyme and a Metal into the Compartments of Mesoporous Silica for Cooperative Tandem Catalysis: An Artificial Metalloenzyme**

*Karin Engström, Eric V. Johnston, Oscar Verho, Karl P. J. Gustafson, Mozaffar Shakeri, Cheuk-Wai Tai, and Jan-E. Bäckvall**

anie_201306487_sm_miscellaneous_information.pdf

General Information

Unless otherwise noted, all materials, reagents and solvents were obtained from commercial suppliers and used without further purification. Dry toluene were column-dried directly before use by a VAC:Solvent Purifier. Aquatic solutions of CALB were provided by either Novozymes A/S (CALB-L) or Fermentas Biotech Limited. Before use, the enzyme was concentrated (to an end concentration of 50 mg/ml) in phosphate buffer (100 mM, pH 8.0) using an Amicon Ultra 10K centrifugal column. The Au nanoparticle colloidal solution used for tagging of CALB was purchased from BBI Life Science (2 nm Au nanoparticles, 20 mL, 249 nmol/L and 5 nm Au nanoparticles, 20 mL, 83 nmol/L Au nanoparticles). GC analysis was done on a Varian 3800 gas chromatograph with a CP-Chirasil-DEX CB column (25 m × 0.32 mm × 0.25 μm). The heterogeneous catalysts were characterized for Pd and enzyme content by Inductively Coupled Plasma (ICP-OES, Medac Ltd, Analytical and Chemical Consultancy Services, United Kingdom). Characterization of the Au and Pd nanoparticles in the various samples were performed by either transmission electron microscopy (TEM) and/or high-angle annular dark-field scanning transmission electron microscopy (HAADF-STEM). TEM and HAADF-STEM images were obtained by a Gatan Ultrascan 1000 camera and a JEOL annular dark-field (ADF) detector, respectively, in a JEOL JEM-2100F 200 kV microscope with ultra-high resolution pole piece (C_s = 0.5 mm; C_c = 1.1 mm). In the HAADF-STEM study, the probe size used was 0.2 nm with the convergence semi-angle of 12.5 mrad and the camera length was 8 cm, which corresponds to the inner and maximum outer semi-angle of the ADF detector of 70 and 180 mrad, respectively. All images were unprocessed.

Procedure Information

Preparation of Pd(0)-AmP-MCF

The amino-functionalized MCF (AmP-MCF) material used for the synthesis of the Pd nanocatalyst, displayed the following characterstics; average pore size = 26-27 nm, window size = 13-14 nm, specific pore volume = 1.65 cm^3/g, and BET surface area = 380 m^2/g. The physical properties of the AmP-MCF material were obtained from N_2 adsorption and desorption isotherms using the Barrett-Joyner-Halenda (BJH) method.

To a suspension of AmP-MCF (1.89 wt% N, 0.50 g) in deionized H_2O (pH 9.0, 20 mL), was added Li_2PdCl_2 (0.22 g, 0.82 mmol) in deionized H_2O (pH 9, 5 mL). The reaction was stirred at room temperature for 24 h, after which the Pd(II)-precatalyst was separated by centrifugation and washed with deionized H_2O (5 × 45 mL). The Pd(II)-precatalyst was then suspended in deionized H_2O (20 mL), and $NaBH_4$ (0.32 mg, 8.46 mmol) in deionized H_2O (5 mL) was added to reduce it to Pd(0). The reduction was performed at room temp for 30 min, after which the Pd nanocatalyst was isolated by centrifugation and washed with deionized H_2O (3 × 45 mL) and acetone (3 × 45 mL). The Pd content of the catalyst was determined by elemental analysis (MedacLtd, ICP-OES), to 7.91 wt%.

Glutaraldehyde functionalization of Pd(0)-AmP-MCF

Pd(0)-AmP-MCF (7.91 wt% Pd, 0.40 g) was suspended in potassium phosphate buffer (25 mL, 100 mM, pH 8.0) and stirred for 5 min in a round-bottomed flask. Glutaraldehyde (50% in H_2O, 0.18 g, 0.78 mmol) was added and the reaction was stirred at room temperature for 24 h. The functionalized Pd catalyst was then isolated by centrifugation, washed with phosphate buffer (3 × 45 mL, 100 mM, pH 8.0) and acetone (3 × 45 mL), and finally dried under vacuum overnight.

Glutaraldehyde functionalization of AmP-MCF

AmP-MCF (7.91 wt% Pd, 0.50 g) was suspended in potassium phosphate buffer (30 mL, 100 mM, pH 8.0) and stirred for 5 min in a round-bottomed flask. Glutaraldehyde (50% in H_2O, 0.23 g, 0.99 mmol) was added and the reaction was stirred at room temp for 24 h. The functionalized MCF-material was then isolated by centrifugation, washed with phosphate buffer (3 x 45 mL, 100 mM, pH 8.0) and acetone (3 × 45 mL), and finally dried under vacuum overnight.

Immobilization of CALB onto glutaraldehyde-functionalized Pd(0)-AmP-MCF

Glutaraldehyde-functionalized Pd(0)-AmP-MCF was stirred overnight in potassium phosphate buffer (1 ml/100 mg support, 100 mM, pH 7.2) with a 50 mg/mL solution of CALB (0.11 mL or

0.34 mL per 100 mg support to give 5.50 mg (low) or 17.0 mg (high) of enzyme, respectively, per 100 mg support). The reaction mixture was then transferred to a glass vial (6 mL), which was placed in a Falcon tube and centrifuged. The supernatant was removed and the precipitated catalyst was washed with potassium phosphate buffer (2 × 5 mL, 100 mM, pH 7.2). The catalyst was then dried under reduced pressure, and stored over a saturated solution of LiCl before use to adjust the water activity to 0.11. The Pd content and CALB content of the hybrid catalysts were determined by ICP-OES analysis and is summarized in Table S12.

Immobilization of CALB onto glutaraldehyde-functionalized AmP-MCF

Glutaraldehyde-functionalized MCF was stirred overnight in a potassium phosphate buffer (1 ml/100 mg support, 100 mM, pH 7.2) with a 50 mg/mL solution of CALB (0.40 ml per 100 mg support to give 20 mg of enzyme per 100 mg support). The reaction mixture was then transferred to a glass vial (6 mL) which was placed in a Falcon tube and was centrifuged. The reaction mixture was then centrifuged, the supernatant was removed and the resulting catalyst was washed with potassium phosphate buffer (2 × 5 mL, 100 mM, pH 7.2). The catalyst was then dried under reduced pressure, and stored over a saturated solution of before use LiCl to adjust the water activity to 0.11.

Procedure for the kinetic resolution of 1-phenylethylamine using CALB immobilized on glutaraldehyde-functionalized AmP-MCF

1-Phenylethylamine (0.60 mmol), ethylmethoxy acetate (1.20 mmol), Na_2CO_3 (50 mg) and CALB-MCF (20.0 mg) were dissolved in toluene (2 mL) and stirred at 70 °C for 1 h under air. Conversion of the kinetic resolutions was determined from the *ee* of the product and the substrate using the equations developed by Sih *et. al.*[1] The results from these experiments are summarized in Table S9.

Procedure for the kinetic resolution of 1-phenylethylamine using the hybrid catalysts

1-Phenylethylamine (0.60 mmol), ethylmethoxy acetate (1.20 mmol), Na_2CO_3 (50 mg) and CALB-MCF (20 mg) were dissolved in toluene (2 mL) and stirred at 70 °C for 1 h under air.

Conversion of the kinetic resolutions was determined from the *ee* of the product and the substrate using the equations developed by Sih *et. al.*[1] The results from these experiments are summarized in Table S8.

Racemization study involving the Pd(0)-AmP-MCF and the hybrid catalysts

(*R*)-(+)-1-phenylethylamine (0.60 mmol) and Pd catalyst (6 µmol) were suspended in dry toluene (4 mL), in a screw-necked Radleys carousel tube. The tube was evacuated and filled with nitrogen two times, before it was evacuated one final time and filled with hydrogen gas. A hydrogen replacement balloon was connected to the tube, and the reaction was stirred at 80 °C. The reaction was sampled periodically by removal of aliquots for GC-analysis by a syringe. The aliquots were derivatized with triethylamine and acetic anhydride prior to GC-analysis to allow for determination of the *ee*. The results from these experiments are shown in Figures S2-7 and Tables S1-6. Racemization experiments employing enzyme-free hybrids were carried out under identical condition and these results are given in Table S7.

Procedure for dynamic kinetic resolution of 1-phenyletylamine using hybrid-$GA_{0.1}$-E_{high}

To a flame dried flask, dry Na_2CO_3 (50 mg) and hybrid-$GA_{0.1}$-E_{high} (30 mg, 15.6 wt% CALB, 4.80 wt% Pd) were added and the flask was evacuated and filled with nitrogen two times, before being refilled with hydrogen gas. Toluene (2 mL) was added and the mixture was heated to 70 °C. Pentadecane (0.5 M in toluene), ethylmethoxy acetate (1.20 mmol) and 1-phenylethylamine (0.60 mmol) were subsequently added. The reaction was then mounted with a hydrogen balloon and allowed to stir for an appropriate time. The *ee* as well as the conversion was monitored periodically using chiral GC. The results from these experiments are summarized in Table 1 (main article).

Procedure for the recycling of the dynamic kinetic resolution of 1-phenylethylamine using Hybrid-$GA_{0.1}$-E_{high}

To a flame dried flask dry Na_2CO_3 (50 mg) and hybrid-$GA_{0.1}$-E_{high} (30 mg, 15.6 wt% CALB, 4.80 wt% Pd) were added and the flask was evacuated and filled with nitrogen two times, before

being refilled with hydrogen gas. Toluene (2 mL) was added and the mixture was heated to 70 °C. Pentadecane (0.5 M in toluene), ethylmethoxy acetate (1.20 mmol) and 1-phenylethylamine (0.60 mmol) was subsequently added, the reaction was mounted with a hydrogen balloon and allowed to stir for the appropriate time. The *ee* and the conversion were monitored by chiral GC. After the reaction reached completion, the mixture was diluted to a total volume of toluene (5 mL) and centrifuged at 4100 rpm for 8 min. The supernatant was removed, toluene (5 mL) was added, and the mixture was centrifuged. This procedure was repeated once. The washed catalyst was then dried by the use of a vacuum pump for 16 h and then placed in a desiccator containing a saturated solution of LiCl for 24 h. The catalyst was then reused under the same conditions as those used above in up to two consecutive cycles. The results from these experiments are summarized in Table S10.

Procedure for the recycling of the kinetic resolution of 1-phenylethylamine using CALB immobilized on glutaraldehyde-functionalized AmP-MCF.

To a flame dried flask dry Na_2CO_3 (50 mg) and CALB-MCF (20 mg, 17 wt% CALB) were suspended in toluene (2 mL) and the flask was evacuated and filled with nitrogen two times. Ethylmethoxy acetate (1.20 mmol) and 1-phenylethylamine (0.60 mmol) was subsequently added and the reaction was stirred at 70 °C. After 1 h the conversion from the kinetic resolution was determined from the *ee* of the product and the substrate using the equations developed by Sih et. al.[1] The results from these experiments are summarized in Table S11. When the reaction had stirred for 24 h, the mixture was diluted to a total volume of toluene (5 mL) and centrifuged at 4100 rpm for 8 min. The supernatant was removed and toluene (5 mL) was added and centrifuged, and this was repeated once. The washed catalyst was then dried by the use of a vacuum pump for 16 h, and then the catalyst was reused under the same condition outlined above in up to two consecutive cycles.

Procedure for dynamic kinetic resolution of 1-phenylethylamine using separate components

To a flame dried flask were added dry Na_2CO_3 (50 mg), CALB-MCF (28 mg, 17 wt% CALB) and Pd-MCF (18 mg, 7.91 wt%). The flask was evacuated and filled with nitrogen twice and

finally refilled with hydrogen gas. Toluene (2 mL) was added and the mixture was heated to 70 °C. Pentadecane (0.5 M in toluene), ethylmethoxy acetate (1.20 mmol) and 1-phenylethylamine (0.6 mmol) were subsequently added. The reaction was then mounted with a hydrogen balloon and allowed to stir for an appropriate time. The *ee* and the conversion were monitored periodically using chiral GC. The results from these experiments are summarized in Table 1 (main article).

TCEP-reduction of a disulfide bridge on the surface on CALB

CALB (80 µL of a 50 mg/mL solution in 100 mM potassium phosphate buffer, pH 8.0) was further diluted with potassium phosphate buffer (200 µL, 100 mM, pH 8.0, 3mM EDTA). To this solution, TCEP (80 µL, 3mM solution in 100mM potassium phosphate buffer, pH 8.0, 3 mM EDTA) was added and the reaction was stirred overnight at room temperature. Thereafter, it was further diluted to 15 mL with potassium phosphate buffer (100 mM, pH 8.0, 3 mM EDTA, 150 mM NaCl). The sample was then concentrated in membrane centrifugation tube with a cutoff of 10K. Dilution and centrifugation were thereafter repeated twice. The concentration of the resulting solution containing the enzyme was determined to 5.5 mg/ml by Bradford protein assay.[2] The extent of disulfide bridge reduction was analyzed by a reaction with Ellman's reagent, which confirmed quantitative reduction (>99%).

Complexation of 2 nm Au nanoparticles onto the surface of CALB and immobilization of the resulting complex onto glutaraldehyde-functionalized Pd(0)-AmP-MCF

The TCEP-reduced CALB (4.8 µL of a 5.5 mg/mL solution) was added to a colloidal solution of 2 nm Au nanoparticles (4.0 mL, molar particle concentration = 249 nM), giving a CALB:Au nanoparticle molar ratio of 1:1.2. The resulting suspension was stirred overnight at room temperature, to afford Au nanoparticle-tagged CALB. The gold nanoparticles that were left unbound in solution were separated from the CALB-Au complexes by the use of a membrane centrifugation tube with a cutoff of 10K (2 nm particles are small enough to pass through the pores). The resulting solution was washed twice with potassium phosphate buffer (2 × 0.5 mL, 100 mM, pH 7.2), and then concentrated to an end volume of 30 µL. The resulting solution was

transferred to a glass vial containing glutaraldehyde-functionalized Pd(0)-AmP-MCF (0.2 mg), and after stirring overnight, the mixture was centrifuged, the supernatant was removed and the precipitated catalyst was washed with potassium phosphate buffer (2 × 0.5 mL, 100 mM, pH 7.2). The catalyst was then dried under reduced pressure.

Complexation of 5 nm Au nanoparticles onto the surface of CALB and immobilization of the resulting complex onto glutaraldehyde-functionalized Pd(0)-AmP-MCF

The TCEP-reduced CALB (4.8 µL of a 5.5 mg/mL solution) was added to a colloidal solution of 5 nm Au nanoparticles (10 mL, molar particle concentration = 83 nM), giving a CALB:Au nanoparticle molar ratio of 1:1. The resulting suspension was stirred overnight at room temperature, to afford Au nanoparticle-tagged CALB. The mixture was then concentrated in a membrane centrifugation tube with a cutoff of 10K to an end volume of 30 µL. The resulting solution was transferred to a glass vial containing glutaraldehyde-functionalized Pd(0)-AmP-MCF (0.2 mg), and after stirring overnight, the mixture was centrifuged, the supernatant was removed and the precipitated catalyst was washed with potassium phosphate buffer (2 × 0.5 mL, 100 mM, pH 7.2). The catalyst was then dried under reduced pressure.

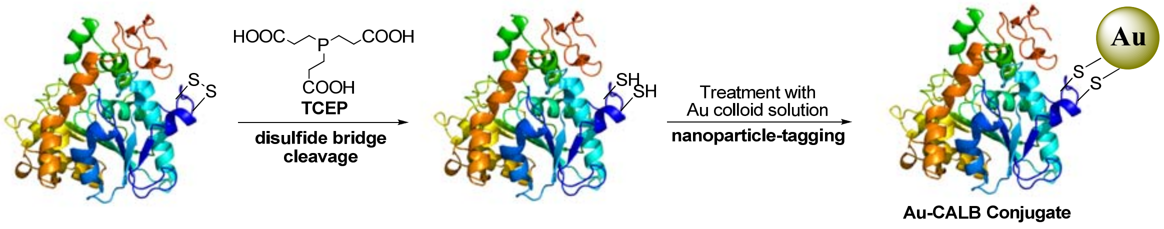

Figure S1. Schematic illustration of the strategy for preparing Au nanoparticle-tagged CALB for indirect enzyme detection by TEM.

Supporting Figures

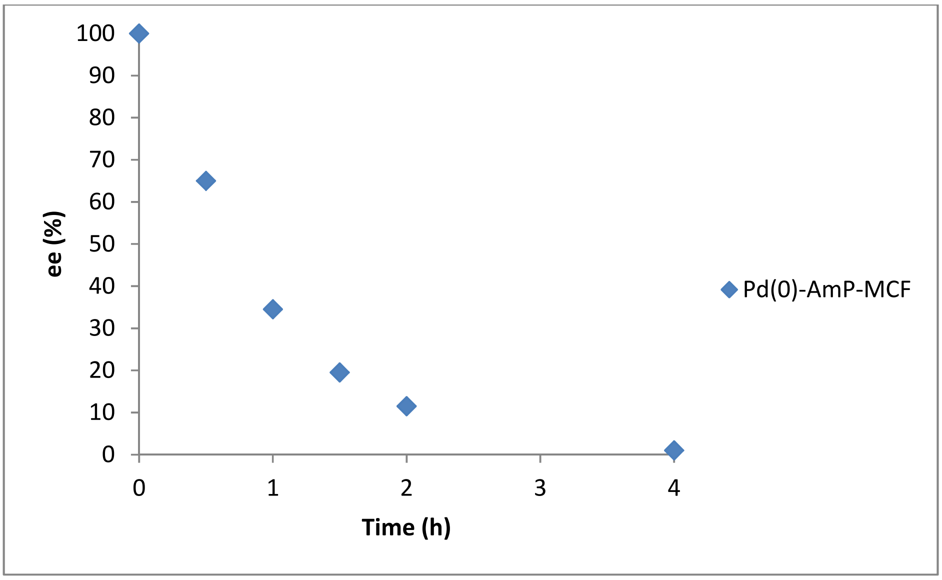

Figure S2. Racemization of (*R*)-(+)-1-phenylethylamine catalyzed by the Pd(0)-AmP-MCF. Only *ee* (mean value of duplicates) is plotted against time (h). Raw data for the graph is given in Table S1.

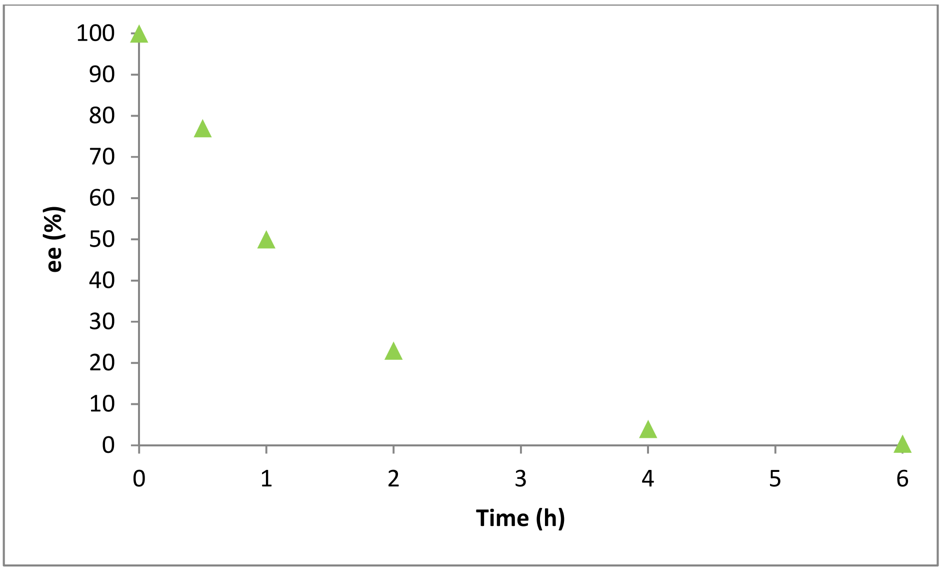

Figure S3. Racemization of (*R*)-(+)-1-phenylethylamine catalyzed by hybrid-$GA_{0.1}$-E_{high}. Only *ee* (mean value of duplicates) is plotted against time (h). Raw data for the graph is given in Table S2.

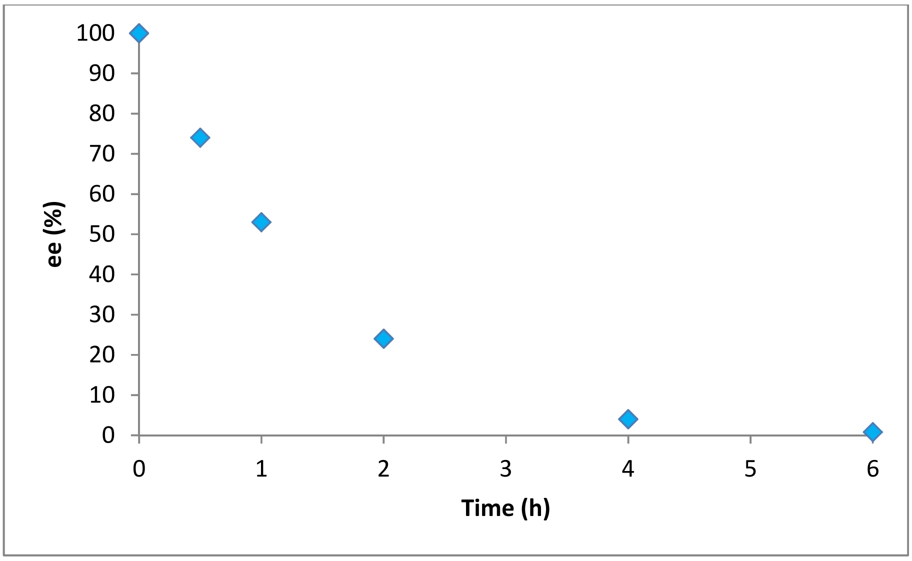

Figure S4. Racemization of (*R*)-(+)-1-phenylethylamine catalyzed by hybrid-$GA_{0.1}$-E_{low}. Only *ee* (mean value of duplicates) is plotted against time (h). Raw data for the graph is given in Table S3.

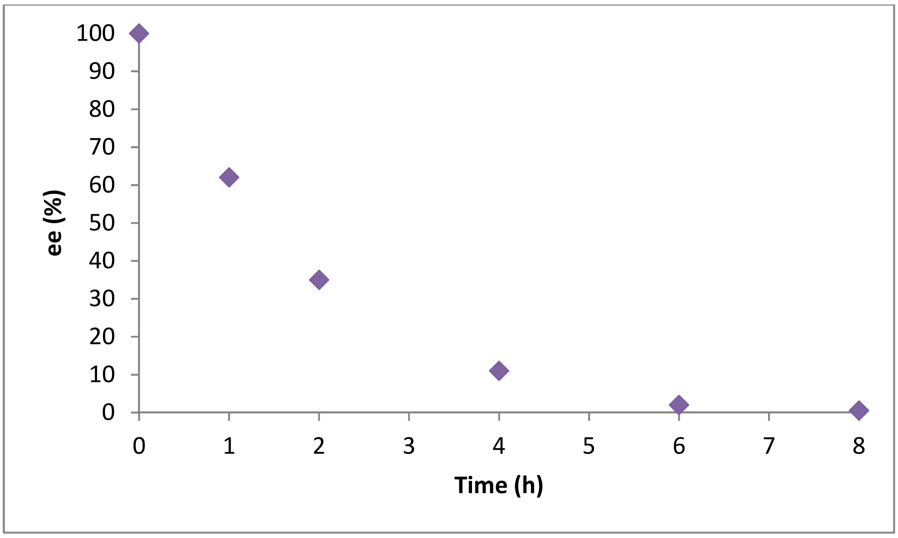

Figure S5. Racemization of (*R*)-(+)-1-phenylethylamine catalyzed by the hybrid-$GA_{0.5}$-E_{high}. Only *ee* (mean value of duplicates) is plotted against time (h). Raw data for the graph is given in Table S4.

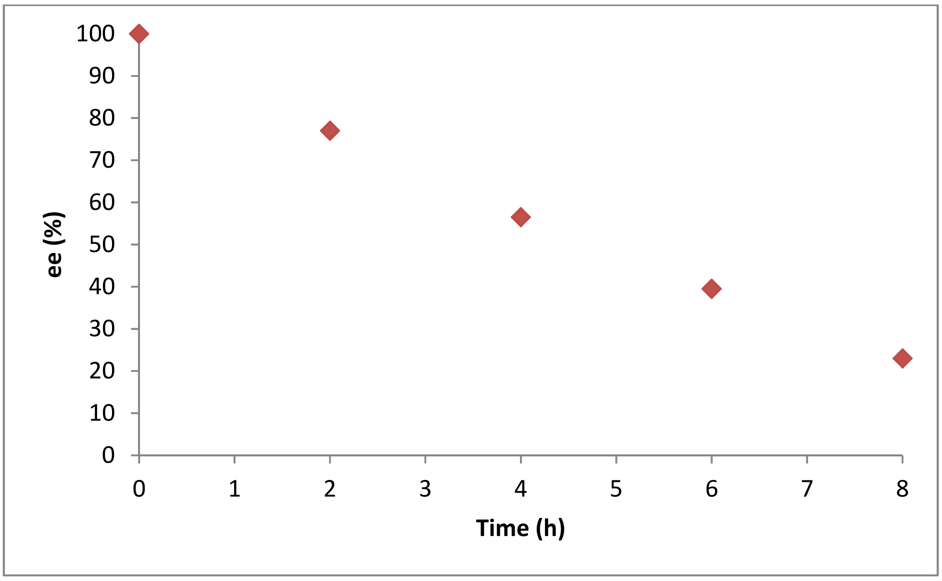

Figure S6. Racemization of (*R*)-(+)-1-phenylethylamine catalyzed by the hybrid-$GA_{2.0}$-E_{high}. Only *ee* (mean value of duplicates) is plotted against time (h). Raw data for the graph is given in Table S5.

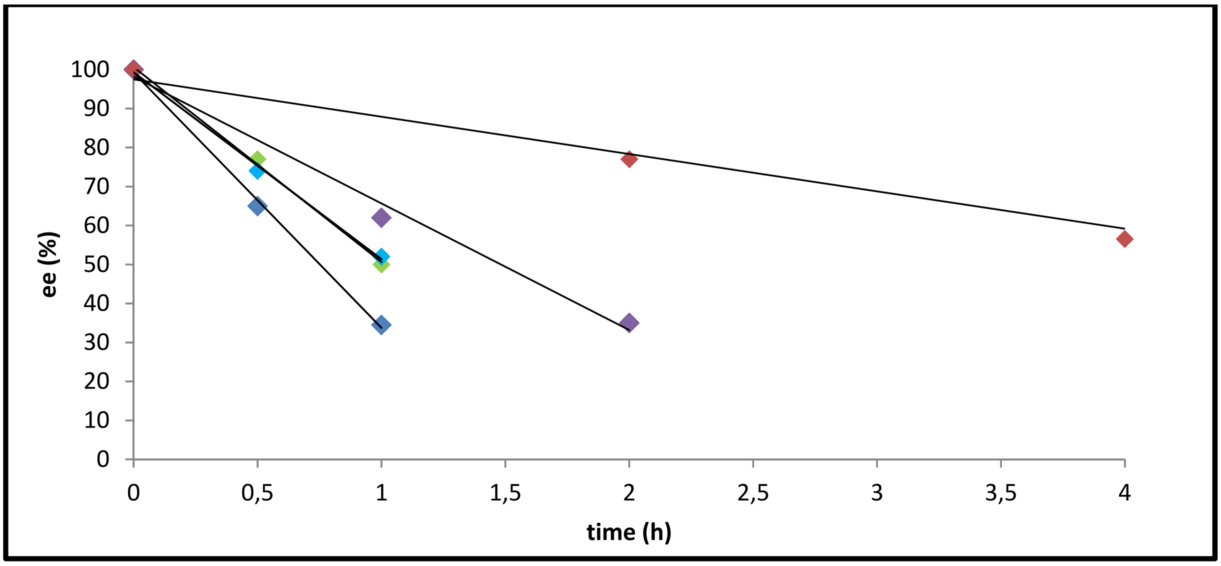

Figure S7. Comparison of the rate of the racemization of (*R*)-(+)-1-phenylethylamine catalyzed by the Pd(0)-AmP-catalyst and the various hybrids prepared in this study (compilation of data from Figures S2-6). For clarity reasons, only the linear regimes of the graphs have been included in this graph. Labels: (♦) hybrid-$GA_{2.0}$-E_{high} (♦) hybrid-$GA_{0.5}$-E_{high} (♦) hybrid-$GA_{0.1}$-E_{low} (▲) hybrid-$GA_{0.1}$-E_{high} (♦) Pd(0)-AmP-MCF.

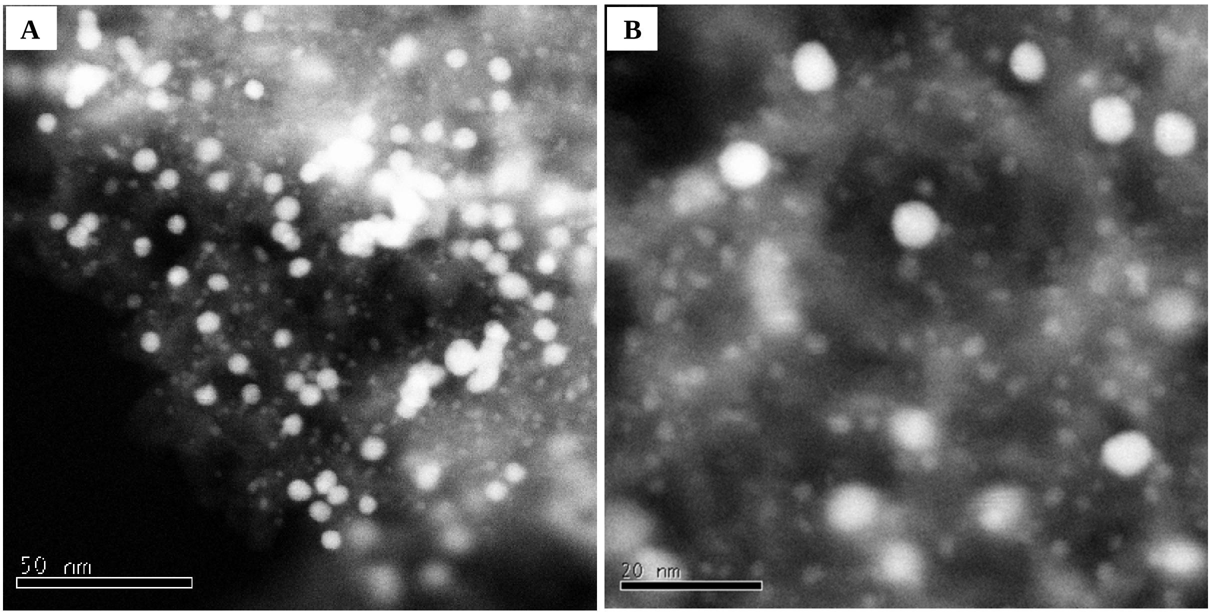

Figure S8. Additional images taken by HAADF-STEM of hybrid-$GA_{0.1}$-E_{high} with Au nanoparticle tags, clearly shows two set of metal nanoparticles. The large 5 nm nanoparticles belongs to Au, while the smaller set on about 1-2 nm belongs to Pd. A) 50 nm scale bar. B) 20 nm scale bar.

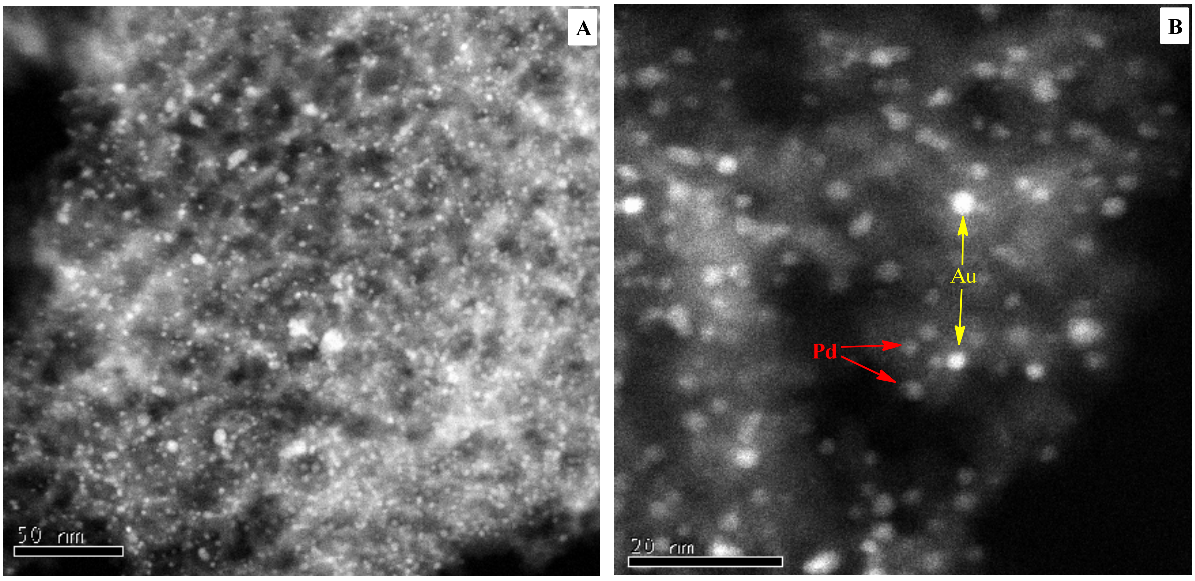

Figure S9. Images taken by HAADF-STEM of hybrid-GA$_{0.1}$-E$_{high}$ with 2 nm Au nanoparticle tags. Differentiation of Au and Pd nanoparticles could not be performed based on the particles size and was instead done by comparing the contrast. In HAADF-STEM, the sensitivity is dependent on the atomic number (Z) of the nanoparticle, making Au (Z = 79) appear with a high contrast than Pd (Z = 46). A) 50 nm scale bar. At this image magnification, it is mostly the Au nanoparticles with sharp contrasts that can be observed, and they exhibit a well-dispersed pattern across the support. B) 20 nm scale bar. The zoomed-in picture allows for an explicit view of the two different sets of nanoparticles and clearly shows their difference in contrast.

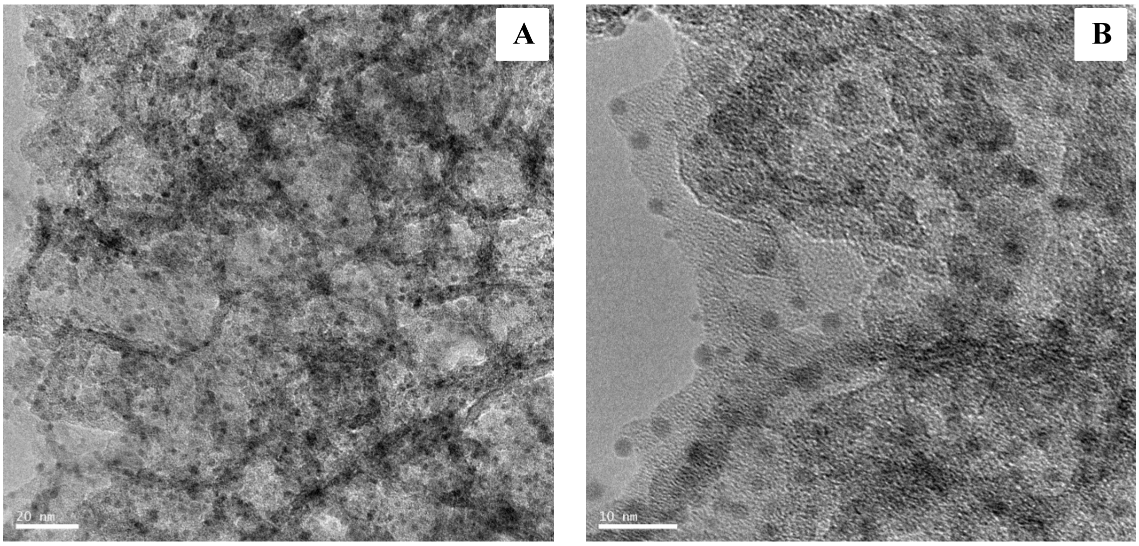

Figure S10. Additional images taken by TEM of hybrid-$GA_{0.1}$-E_{high} without Au nanoparticle tags, shows well-dispersed Pd nanoparticles in the size range of 1-2 nm. A) 20 nm scale bar. B) 10 nm scale bar.

Supporting Tables

Table S1. Raw data for the racemization of (*R*)-(+)-1-phenylethylamine catalyzed by the Pd(0)-AmP-MCF (Figure S2).

t (h)	*ee* (%) 1	*ee* (%) 2	*ee* (mean)
0	100	100	100
0.5	60	70	65
1.0	34	35	34.5
1.5	20	19	19.5
2.0	11	12	11.5
4.0	1	1	1

Table S2. Raw data for the racemization of (*R*)-(+)-1-phenylethylamine catalyzed by hybrid-$GA_{0.1}$-E_{high} (Figure S3)

t(h)	*ee* (%) 1	*ee* (%) 2	*ee* (mean)
0	100	100	100
0.5	74	80	77
1.0	48	52	50
2.0	20	25	22.5
4.0	3	5	4
6.0	0.3	0.5	0.4

Table S3. Raw data for the racemization of (*R*)-(+)-1-phenylethylamine catalyzed by hybrid-$GA_{0.1}$-E_{low} (Figure S4).

t(h)	*ee* (%) 1	*ee* (%) 2	*ee* (mean)
0	100	100	100
0.5	71	77	74
1.0	52	54	52
2.0	22	25	23.5
4.0	3	5	4
6.0	0.6	1.0	0.8

Table S4. Raw data for the racemization of (*R*)-(+)-1-phenylethylamine catalyzed by the hybrid-$GA_{0.5}$-E_{high} (Figure S5).

t(h)	*ee* (%) 1	*ee* (%) 2	*ee* (mean)
0	100	100	100
1	58	66	62
2	33	37	35
4	10	12	11
6	1.5	2.5	2
8	0.5	0.5	0.5

Table S5. Raw data for the racemization of (*R*)-(+)-1-phenylethylamine catalyzed by the hybrid-$GA_{2.0}$-E_{high} (Figure S6).

t (h)	*ee* (%) 1	*ee* (%) 2	*ee* (mean)
0	100	100	100
2	79	75	77
4	60	53	56.5
6	44	35	39.5
8	23	23	23

Table S6. Data obtained from Figure S7.

Catalyst	Slope (initial rate)	Relative rate	R^2
Pd(0)-AmP-MCF	-66.5	1	0.998
Hybrid-$GA_{0.1}$-E_{high}	-50.0	0.75	0.998
Hybrid-$GA_{0.1}$-E_{low}	-48.5	0.73	0.998
Hybrid-$GA_{0.5}$-E_{high}	-32.5	0.49	0.991
Hybrid-$GA_{2.0}$-E_{high}	-10.9	0.16	0.999

Table S7. Racemization of (*R*)-(+)-1-phenylethylamine by enzyme-free Pd(0)-AmP-MCF with varying support of glutaraldehyde

Catalyst	*ee* (mean) 30 min	*ee* (mean) 1h	*ee* (mean) 2h
Pd(0)-AmP-MCF $GA_{0.1}$	79	64	40
Pd(0)-AmP-MCF $GA_{0.5}$	86	71	51
Pd(0)-AmP-MCF $GA_{2.0}$	98	96	94

Table S8. Kinetic Resolution of 1-phenylethylamine by hybrid catalysts.[a]

Entry	Hybrid	ee_p	ee_s	Conv.[b]	E-value[b]
1	$GA_{0.1}$-E_{high}	99	56	36	>200
2	$GA_{0.1}$-E_{low}	99	21	18	>200
3	$GA_{0.5}$-E_{high}	99	56	36	>200
4	$GA_{2.0}$-E_{high}	99	66	40	>200

a) Reaction conditions: 1-phenylethylamine (0.60 mmol), ethylmethoxy acetate (1.20 mmol), Na_2CO_3 (50 mg) and hybrid catalyst (10 mg) were dissolved in toluene (1 mL) and stirred at 70°C for 1 h. b) Determined from ee_s and ee_p by the use of the equations by Sih *et. al.*

Table S9. Kinetic Resolution of 1-phenylethylamine by CALB- MCF.[a]

Entry	Temp (°C)	Time (h)	ee_p	Conv.[b]	E-value[b]
1	70	1.0	99	39	>200
2	80	0.5	99	35	>200
		1.0	99	45	>200

a) Reaction conditions: 1-phenylethylamine (0.60 mmol), ethylmethoxy acetate (1.20 mmol), Na_2CO_3 (50 mg) and CALB-MCF (10 mg) were dissolved in toluene (1 mL) and stirred for the temperature and time given in the table. b) Determined from ee_s and ee_p by the use of the equations by Sih *et. al.*

Table S10. Recycling study of the hybrid-$GA_{0.1}$-E_{high} in the DKR of 1-phenylethylamine with and without molecular sieves (4Å).[a]

Entry	Cycle	Time [h]	Yield [%][b]	ee [%][b]
1	1	16	95	99
2	2	16	68	99
		24	88	99
		48	96	99
3	3	72	42	99
4[c]	1	16	99	99
5[c]	2	16	74	99
		48	82	99

[a] Reaction conditions: All the reactions were carried out in toluene under 1 atm of hydrogen gas at 70°C, 1-phenylethylamine (0.60 mmol), ethyl methoxy acetate (1.20 mmol), dry Na_2CO_3 (50 mg), and pentadecane as internal standard. [b] Determined by GC-analysis [c] Performed with molecular sieves (4 Å).

Table S11. Recycling study of palladium-free CALB-MCF in kinetic resolution of 1-phenylethylamine [a]

Cycle	Time [h]	Yield [%][b]	ee [%][b]
1	1	36	99
2	1	30	99
3	1	16	99

Reaction conditions: All the reactions were carried at 70°C, 1-phenylethylamine (0.60 mmol), ethyl methoxy acetate (1.20 mmol), dry Na_2CO_3 (50 mg), and pentadecane as internal standard.[b] Determined by GC analysis.

Table S12. Determination of the Pd and CALB content by elemental analysis (ICP-OES)

Hybrid	CALB wt%	Pd wt%
$GA_{0.1}$-E_{high}	15.6	4.80
$GA_{0.1}$-E_{low}	5.20	5.89
$GA_{0.5}$-E_{high}	14.4	4.61
$GA_{2.0}$-E_{high}	17.0	5.78

References

1. C. -S. Chen, Y. Fujimoto, G. Girdaukas, C. J. Sih, *J. Am. Chem. Soc.* **1982**, *104*, 7294.
2. M. M. Bradford, *Anal Biochem.* **1976**, *72*, 248.

Representative GC chromatogram for the DKR of 1-phenylethylamine used for *ee* determination (with pentadecane as IS, t_r =8.078 min)

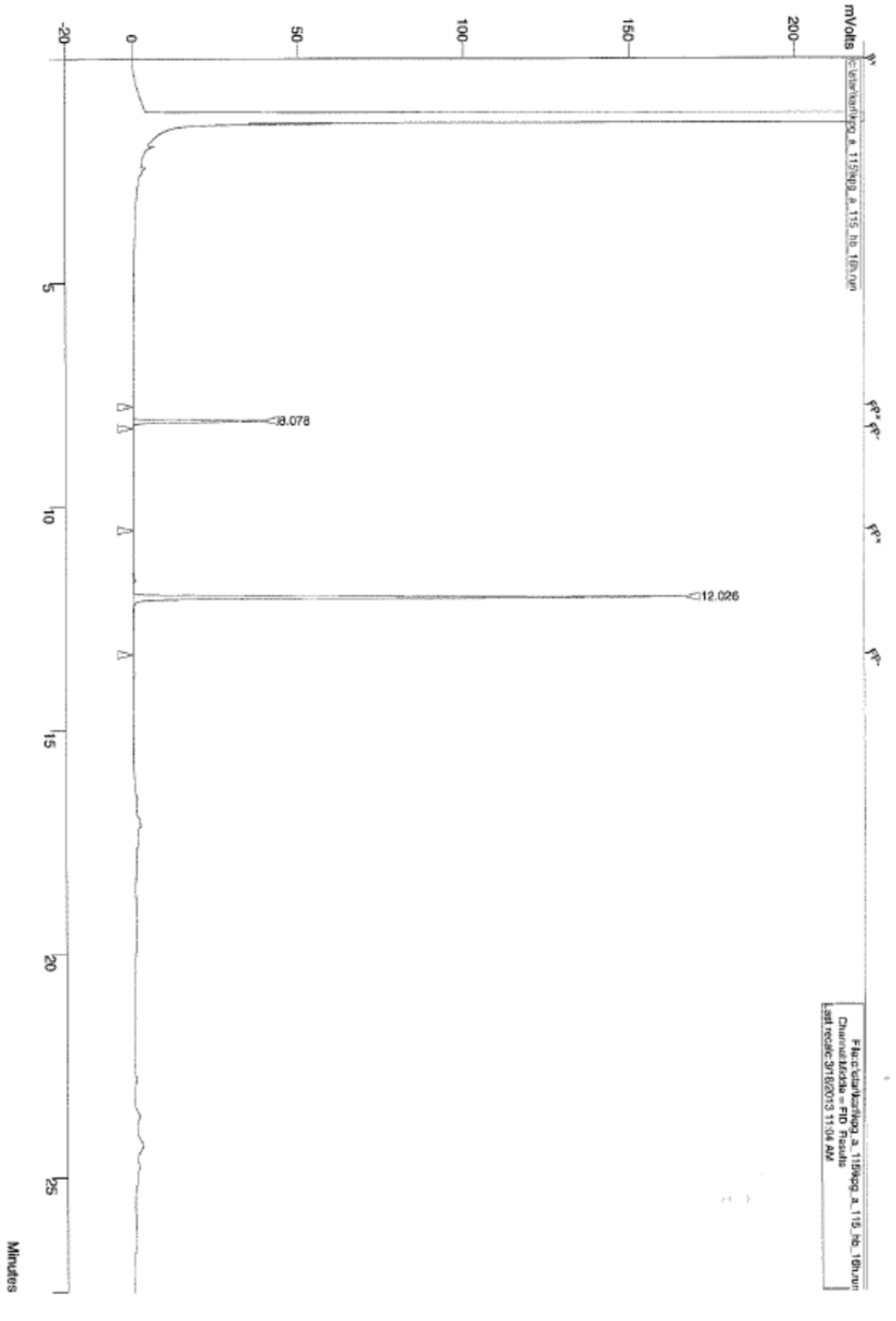

Representative GC chromatogram for racemic 1-phenylethylamine reference

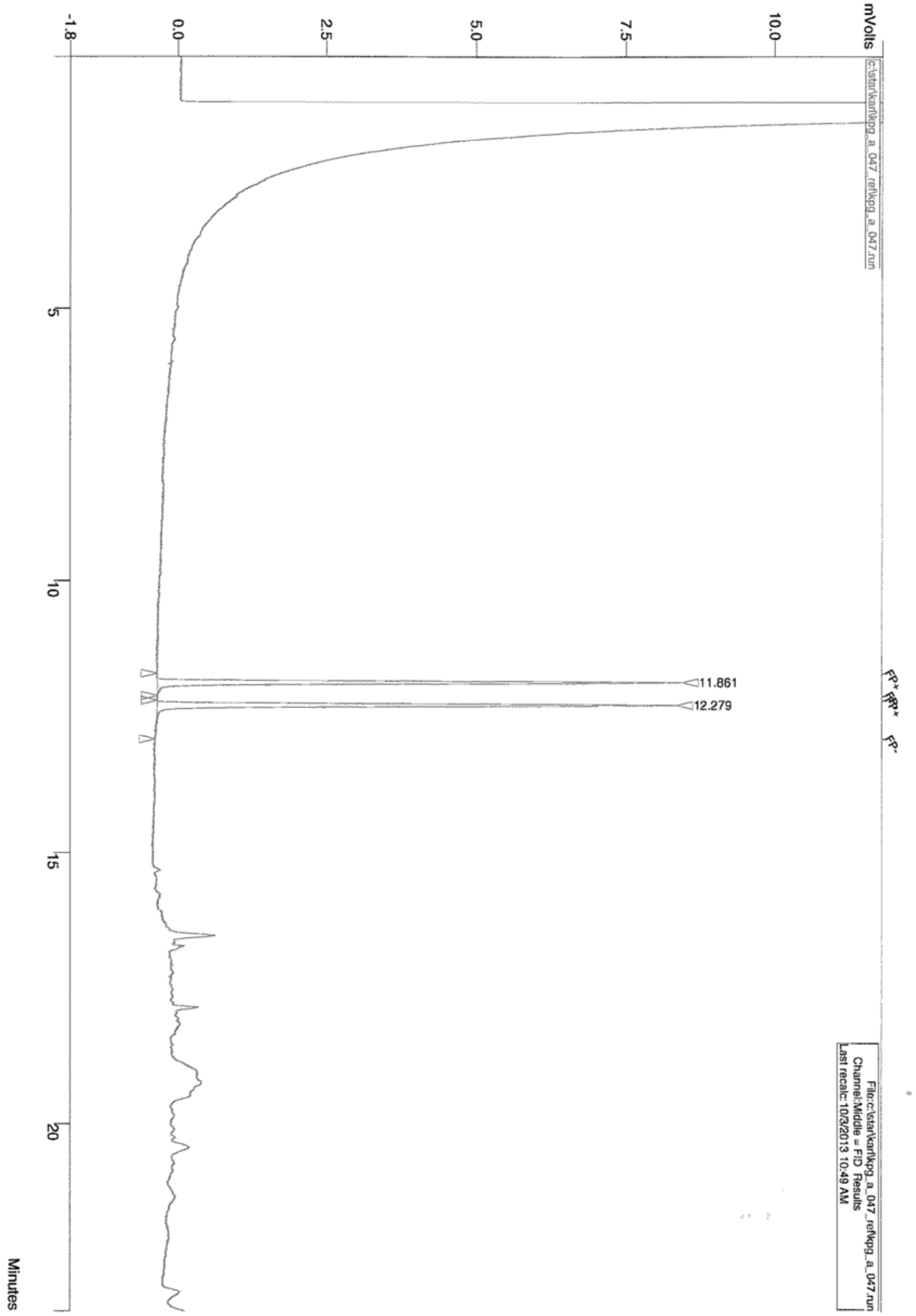

Supplement: Supplementary file 1 [file anie0052-14006-sd1.pdf]
